# Firefly-mimicking intensive and long-lasting chemiluminescence hydrogels

**DOI:** 10.1038/s41467-017-01101-6

**Published:** 2017-10-17

**Authors:** Yating Liu, Wen Shen, Qi Li, Jiangnan Shu, Lingfeng Gao, Mingming Ma, Wei Wang, Hua Cui

**Affiliations:** 1 CAS Key Laboratory of Soft Matter Chemistry, Collaborative Innovation Center of Chemistry for Energy Materials, Department of Chemistry, University of Science and Technology of China, Hefei, Anhui 230026 China; 2 School of Chemistry and Chemical Engineering, State Key Laboratory of Analytical Chemistry for Life Science, Nanjing University, Nanjing, Jiangsu 210093 China

## Abstract

Most known chemiluminescence (CL) reactions exhibit flash-type light emission. Great efforts have been devoted to the development of CL systems that emit light with high intensity and long-lasting time. However, a long-lasting CL system that can last for hundreds of hours is yet-to-be-demonstrated. Here we show firefly-mimicking intensive and long-lasting CL hydrogels consisting of chitosan, CL reagent N-(4-aminobutyl)-N-ethylisoluminol (ABEI) and catalyst Co^2+^. The light emission is even visible to naked eyes and lasts for over 150 h when the hydrogels are mixed with H_2_O_2_. This is attributed to slow-diffusion-controlled heterogeneous catalysis. Co^2+^ located at the skeleton of the hydrogels as an active site catalyzes the decomposition of slowly diffusing H_2_O_2_, followed by the reaction with ABEI to generate intensive and long-lasting CL. This mimics firefly bioluminescence system in terms of intensity, duration time and catalytic characteristic, which is of potential applications in cold light sources, bioassays, biosensors and biological imaging.

## Abstract (web summary)

Great efforts have been devoted to the development of chemiluminescence systems that emit light with high intensity over long periods of time. Here the authors show, firefly-mimicking intensive and long-lasting chemiluminescence hydrogels consisting of chitosan, N-(4-aminobutyl)-N-ethylisoluminol (ABEI) and catalyst Co^2+^.

## Introduction

Light emission induced by chemical reactions, known as chemiluminescence (CL), has been intensively investigated for many years. It has been widely applied in cold light sources, bioassays, reporter genes, biological imaging, and biomapping^1–4^. However, most known CL reactions exhibit flash-type light emissions, which hampers their applications. Light emission with high intensity and long-lasting time, i.e., glow-type emission, has been the holy grail of CL field. For example, strong and long-lasting emission are crucial for cold light sources in emergency situations, decorative entertainment, and underwater lighting. In analytical chemistry fields, the CL reactions with flash-type emission generally carry out CL emission in seconds or minutes and possess a fast kinetic curve, which would lead to poor analytical accuracy. The glow-type emission can produce a slow kinetic curve, even a constant emission within analytical time, which would improve greatly the analytical sensitivity and accuracy^5^. Besides, CL is less often used for imaging than fluorescence due to the lack of strong and long-lasting CL probes^4, 6^. Strong and long-lasting emission is beneficial for the investigation in the field of CL imaging with microscopy. In the past, enzyme-involved CL reactions are main CL systems producing long-lasting emission. For enzymatic reactions such as firefly bioluminescence (BL) system, bacterial BL system, alkaline phosphatase-3-(2′-spiroadamantane)-4-methoxy-4-(3″-phosphoryloxy)phenyl-1,2-dioxetane system and luminol-H_2_O_2_-peroxidases system, long-lasting emission arose from the turnover of the enzymes and excessive substrates^7–9^. However, enzyme inactivation was the main reason for light decay in the above CL systems^10^. Besides, peroxyoxalate ester CL system could also produce long-lasting emission under controlled conditions. The nucleophilic reaction of hydrogen peroxide with peroxyoxalate esters to generate a high energy intermediate dioxetandione is the rate-determining step for the CL reaction. The activated intermediate complex formed by dioxetandione and fluorophore can be continuously produced by succeeding supply of excess oxalate and fluorophore^9^. Since then, no new long-lasting CL mechanism has been discovered for a long time. Intensive and long-lasting CL emission is highly desired for sensitive and accurate bioassays, cold light sources and CL imaging, but is still a great challenge.

Herein, we report an intensive and long-lasting CL chitosan (CS) hydrogel with CL reagent N-(4-aminobutyl)-N-ethylisoluminol (ABEI) and catalyst Co^2+^ (ABEI/Co^2+^/CS hydrogels) by virtue of a slow-diffusion-controlled heterogeneous catalytic mechanism, which mimics the firefly BL system in terms of both catalytic and kinetic characteristics. The light emission is even visible to naked eyes and lasts for over 150 h.

## Results

### Synthesis and characterizations

**Fig. 1:**
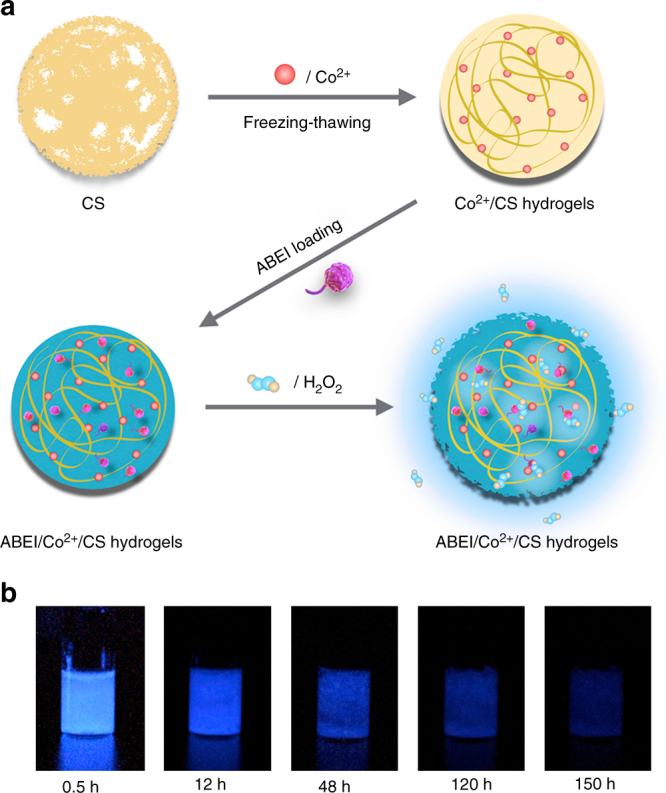
Schematic illustration. **a** Preparation of ABEI/Co^2+^/CS hydrogels. **b** CL emission of ABEI/Co^2+^/CS hydrogels

The preparation of ABEI/Co^2+^/CS hydrogels is shown in Fig. 1a. Initially, chitosan powders dispersed in alkaline solution were mixed with CoCl_2_ solution. Through freezing-thawing process^11^, Co^2+^/CS hydrogels were obtained. Next, ABEI alkaline solution was mixed with Co^2+^/CS hydrogels and stirred to obtain ABEI/Co^2+^/CS hydrogels. The as-prepared hydrogels were characterized by scanning electronic emission (SEM), rheology experiments, inductively coupled plasma atomic emission spectroscopy (ICP-AES) and UV-visible absorption spectra.

**Fig. 2:**
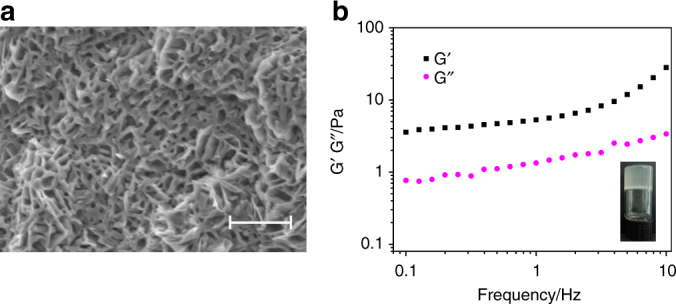
Characterization of Co^2+^/CS hydrogels and ABEI/Co^2+^/CS hydrogels. **a** SEM images of freeze-dried Co^2+^/CS hydrogels with 10-folds dilution. Scale bar is 10 µm. **b** Frequency dependence of dynamic storage modulus (G') and loss modulus (G") of ABEI/Co^2+^/CS hydrogels with 1 % strain at 20 °C. Inset in Fig. 2b: optical image of ABEI/Co^2+^/CS hydrogels

As shown in Fig. 2a, Co^2+^/CS hydrogels (10 times dilution) possessed porous sponge-like structure with lots of micro-sized and even nano-sized pores, which are consistent with those of previously reported CS hydrogel^11, 12^. The porosity of the hydrogels was determined to be 86%. The sponge-like structure with high porosity was endowed with high adsorption capacity for fluids. Moreover, these pores were effective channels for loading small molecules. Therefore, it is reasonable to assume that this porous structure is a wonderful storage of small molecules. The viscoelastic properties of ABEI/Co^2+^/CS hydrogels were studied by rheology experiments. After setting the strain amplitude at 1% (within the linear response of strain amplitude as shown in Supplementary Fig. 1), dynamic frequency sweep of ABEI/Co^2+^/CS hydrogels was carried out. As shown in Fig. 2b, the dynamic storage modulus (G′) and loss modulus (G″) increased with the increase of the frequency from 0.1 to 10 Hz, and G′ was 3-4 times higher than G" at the same frequency. This is consistent with the observation that the materials possessed a gel-like structure that barely flowed (test tubes could be tilted upside down without sample flowing, see inset in Fig. 2b). Moreover, the CS hydrogels formed mainly by physical crosslinking^13^ are translucent, thus they are solvent-incompatible system. This is consistent with the fact that CS has a low solubility in alkaline aqueous solution^14^.

The composition of the hydrogels was characterized. ICP-AES elemental analysis showed the existence of Co. As shown in Supplementary Fig. 2, the characteristic absorption peaks of pure ABEI appeared at around 290 and 320 nm. The Co^2+^/CS hydrogels did not have obvious characteristic absorption peaks. The two characteristic absorption peaks of ABEI at 291 and 320 nm were also observed in the UV/visible spectrum of ABEI/Co^2+^/CS hydrogels, indicating the existence of ABEI molecules in ABEI/Co^2+^/CS hydrogels. Therefore, both ABEI and Co^2+^ were successfully entrapped into the hydrogels. The ABEI and Co^2+^ used for synthesis were all trapped inside the hydrogels because no further separation treatment of the as-prepared hydrogels was conducted after synthesis.

### CL performance

**Fig. 3:**
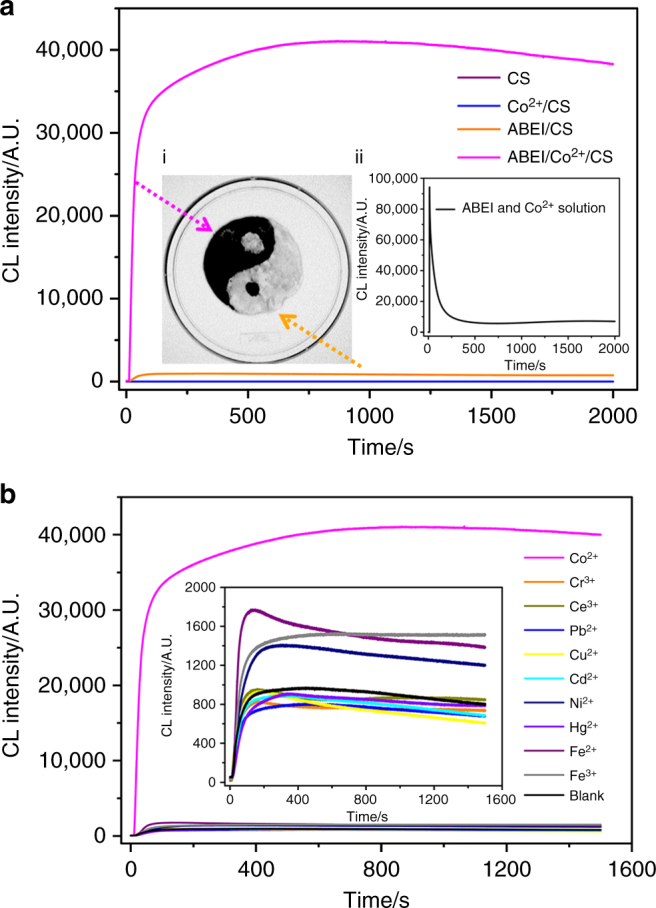
CL performance. **a** CL kinetic curves for reaction of CS hydrogels, Co^2+^/CS hydrogels, ABEI/CS hydrogels, ABEI/Co^2+^/CS hydrogels with H_2_O_2_. Inset (i): gel CL imaging of ABEI/Co^2+^/CS hydrogel-H_2_O_2_ (black) and ABEI/CS hydrogel-H_2_O_2_ (white). Inset (ii): CL kinetic curves for reaction of Co^2+^-ABEI-H_2_O_2_ system with a fixed photomultiplier tube (PMT) voltage of −450 V. **b** CL kinetic curves for reaction of ABEI/metal ion/CS hydrogels using different metal ions (Co^2+^, Cu^2+^, Pb^2+^, Ni^2+^, Hg^2+^, Cr^3+^, Ce^3+^, Cd^2+^, Fe^2+^, Fe^3+^ and blank) with H_2_O_2_. Inset: magnification of Cu^2+^, Pb^2+^, Ni^2+^, Hg^2+^, Cr^3+^, Ce^3+^, Cd^2+^, Fe^2+^, Fe^3+^ and blank (without metal ion) in the ABEI/metal ion/CS hydrogels-H_2_O_2_. Reaction condition: 100 μl 0.1 M H_2_O_2_, 100 μl hydrogels, -550 V PMT

When ABEI/Co^2+^/CS hydrogels reacted with H_2_O_2_ solution, firefly-mimicking intensive and long-lasting CL emission appeared, as shown in Fig. 1b. The light emission could be observed even by naked eyes in a dark room and lasted for over 150 h. The CL kinetic behavior of ABEI/Co^2+^/CS hydrogels was further investigated by static injection as shown in Fig. 3a. 100 μl of the hydrogels was injected into 100 μl of 0.1 M H_2_O_2_ solution. Strong and long-lasting light emission was observed from ABEI/Co^2+^/CS hydrogels (magenta curve), while no light emission from CS hydrogels (purple curve) and Co^2+^/CS hydrogels (blue curve). The CL spectrum of ABEI/Co^2+^/CS hydrogels with H_2_O_2_ exhibited a peak centered at  ~ 440 nm, as shown in Supplementary Fig. 3, which was consistent with that of the CL reaction of ABEI with H_2_O_2_^15^. These results demonstrated that the CL reaction of ABEI with H_2_O_2_ was responsible for the light emission. Co^2+^ was further found to enhance the CL intensity by 40 times when comparing the CL intensities in the presence (magenta curve) and absence (orange curve) of Co^2+^. The CL signal of ABEI/Co^2+^/CS hydrogels was not only strong, but also stable for over 25 min (the CL intensity only decreased to 93% of the maximum value in 25 min). The CL emission was recorded by gel CL imaging as shown in inset (i) of Fig. 3a. Based on the high contrast of ABEI/CS hydrogels and ABEI/Co^2+^/CS hydrogels in CL intensity, traditional Chinese Taiji pattern was painted. ABEI/CS hydrogels showed a weak CL (white part) while ABEI/Co^2+^/CS hydrogels a strong CL (black part). When Co^2+^ and ABEI solutions were directly mixed with H_2_O_2_ in alkaline solution, a flash CL emission was obtained as shown in inset (ii) of Fig. 3a. The CL reaction kinetic of the Co^2+^-ABEI-H_2_O_2_ system is quite different from those of ABEI/Co^2+^/CS hydrogel-H_2_O_2_. The results demonstrated that the formation of hydrogels affected the CL reaction kinetic of Co^2+^- catalyzed ABEI-H_2_O_2_ system and played an important role in intensive and long-lasting CL. The stability of trapped ABEI and the sustainability of the hydrogel system were also studied by determining fluorescence intensity of ABEI and CL intensity of the hydrogels with H_2_O_2_ as functions of time, as shown in Supplementary Figs. 4 and 5. The results demonstrated that trapped ABEI and the hydrogels were stable in at least 30 days.

The proposed long-lasting CL system is compared with other non-enzymatic and enzymatic CL systems. Peroxyoxalate esters and firefly BL are typical non-enzymatic and enzymatic CL systems for long-lasting CL emission, respectively. In the peroxyoxalate ester systems, light emission of bis(6-alkoxycarbonyl-2,4-dichlorophenyl) oxalates could last more than 12 h with low-intensity^16^. In firefly BL, typical emission could last for more than 6 h when firefly luciferase was in live cells and 2 h when firefly luciferase was in solution^17, 18^. The light emission of our CL system was even visible to naked eyes and lasted for over 150 h. Accordingly, our system have distinguished CL intensity and duration time, which superior to non-enzymatic peroxyoxalate ester CL system and enzymatic firefly BL system.

It has been reported that various metal ions could catalyze the CL reactions of luminol and its analogues with H_2_O_2_^19^. Thus, instead of Co^2+^, other metal ions Cu^2+^, Pb^2+^, Ni^2+^, Hg^2+^, Cr^3+^, Ce^3+^, Cd^2+^, Fe^2+^ and Fe^3+^ were used to prepare the CS hydrogels. As shown in Fig. 3b, compared with ABEI/CS hydrogels without any metal ions, the CL intensity using Co^2+^, Fe^3+^, Fe^2+^, Ni^2+^, Ce^3+^, Cu^2+^, Hg^2+^, Cd^2+^, Cr^3+^ and Pb^2+^ changed by 4126, 82, 57, 45, −2, −4, −6, −8, −15, −17%, respectively. ABEI/Co^2+^/CS hydrogels exhibited unique CL enhancement among all the tested metal ions. Other metal ions of Group VIII in periodic table also showed enhancement to some extent and the rest of the aforementioned ions did not significantly affect CL intensity. Therefore, the CL hydrogels using Co^2+^ are optimal for achieving intensive CL emission.

### Optimization for CL conditions

**Fig. 4:**
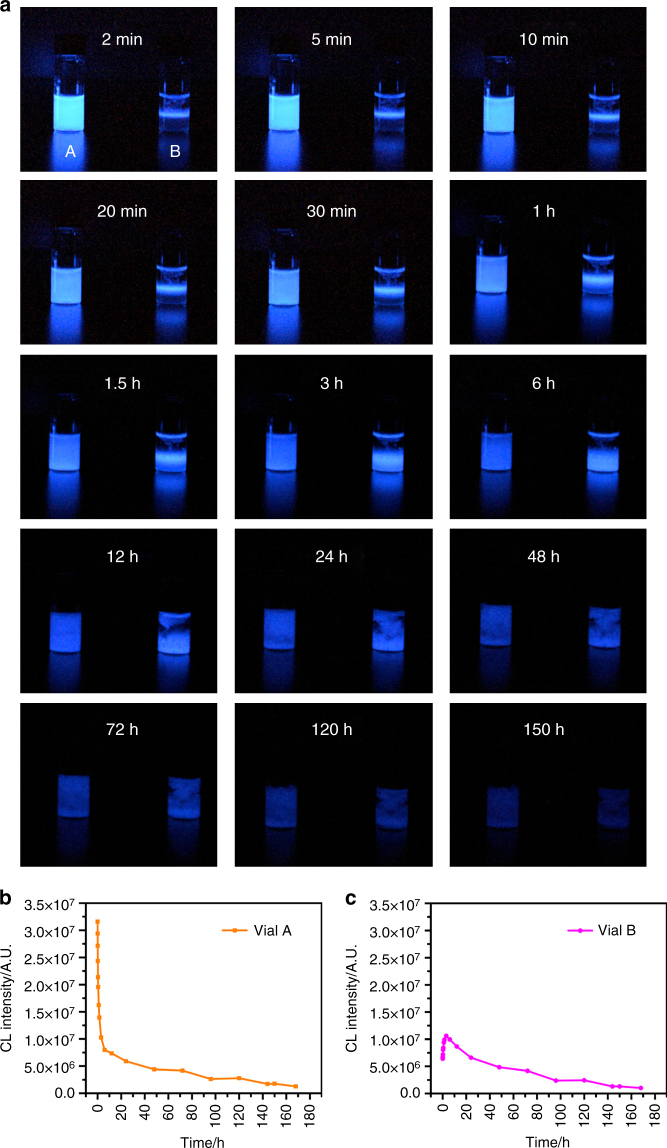
Reaction of ABEI/Co^2+^/CS hydrogels with H_2_O_2_ at different times. **a** Optical images using a digital camera. H_2_O_2_ solution was fully mixed with ABEI/Co^2+^/CS hydrogels in vial A and H_2_O_2_ solution was directly added into ABEI/Co^2+^/CS hydrogels without agitation in vial B. All the images are coded according to the same intensity scale. **b**, **c** CL intensity as a function of time for vial A and B, respectively. For ABEI/Co^2+^/CS hydrogels, 40 mM 1.5 ml ABEI, 1 mM 0.6 ml Co^2+^, 15 ml CS dispersed in alkaline solution. Reaction condition: 1 ml 0.1 M H_2_O_2,_ 1 ml hydrogels

Some important parameters affecting the intensity and duration time of CL emission and the formation of the hydrogels, including the concentration of ABEI and Co^2+^, were studied. As shown in Supplementary Figs. 6 and 7, the CL intensity increased with the increase of ABEI and Co^2+^ concentrations. However, high Co^2+^ concentration was not favored for long-lasting time and hydrogel formation. Besides, it was also found that CL signal was highly dependent on the concentration of H_2_O_2_ (Supplementary Fig. 8). The CL intensity and the duration time increased with increasing the concentration of H_2_O_2_ up to 0.1 M. However, when the concentration of H_2_O_2_ was higher than 0.1 M, the CL intensity was slightly down and the CL was instable. It is possible that the oxygen bubbles due to H_2_O_2_ decomposition had an effect on the stability of the CL reaction. The effect of pH of H_2_O_2_ solution on the CL emission was studied in the pH range of 7.0–13.0. The CL emission could still be observed under neutral conditions. The CL intensities remained almost constant upon increasing pH values, and the optimal time for plateau emission was at pH 10.88 (Supplementary Fig. 9). Under optimal conditions, digital camera was used to record CL emission image in real time when H_2_O_2_ solution was either mixed fully with ABEI/Co^2+^/CS hydrogels (Fig. 4a vial A) or added into ABEI/Co^2+^/CS hydrogels directly (Fig. 4a vial B). Figures 4b, 4c also shows the CL intensity as a function of time over 150 h (the CL intensity was calculated as described in Supplementary Note 1). Both of cases showed intensive emission, which was even visible in a dark room with naked eyes. It is noteworthy that CL could last for more than 150 h. The CL spectra of ABEI/Co^2+^/CS hydrogels with H_2_O_2_ at different times were also measured, as shown in Supplementary Fig. 10. No obvious change in CL spectra was observed.

## Discussion

Such intensive and long-lasting emission of ABEI/Co^2+^/CS hydrogels could be ascribed to the following reasons. As reported, metal ions, such as Co^2+^, could be highly adsorbed by chitosan by forming coordination bonds via hydroxyl and amine groups^20, 21^. Metal ions in the chitosan hydrogels could function as stabilizing linkages to prevent gel dissolution^22^. Thus, it may be suggested that Co^2+^ coordinates with the skeleton of the CS hydrogels and most of Co^2+^ exists in the CS phase. When the ABEI/Co^2+^/CS hydrogels were centrifuged, Co^2+^ and ABEI concentration in supernatant were determined to be 6.41 × 10^−5^ and 3.14 mM, respectively, as shown Supplementary Table 1 and Note 2. Co^2+^ and ABEI concentration in ABEI/Co^2+^/CS hydrogels were 3.51 × 10^−2^ and 3.51 mM, respectively. The results demonstrated that 0.18 % Co^2+^ and 89.48% ABEI existed in aqueous phase. Thus almost all of Co^2+^ was immobilized at the skeleton of the CS hydrogels. The porous network structure possessed micro/nano-sized pores and acted as a water-absorbing sponge, which allowed high concentration of ABEI to be loaded into the pores of the hydrogels. The uniform dispersion of ABEI in the porous network structure of hydrogels was confirmed by the fluorescence imaging of ABEI/Co^2+^/CS hydrogels (Supplementary Fig. 11). Vial B in Fig. 4a shows the real-time reaction which involves adding 1 ml H_2_O_2_ solution onto the top of 1 ml hydrogels without further agitation. At the beginning, the CL reaction merely occurred at the interface between ABEI/Co^2+^/CS hydrogels and H_2_O_2_ solution. Gradually, the H_2_O_2_ diffused into the hydrogels and more CL emission could be seen from the hydrogels. Due to the slow diffusion in hydrogels, it took 3–6 h for the top layer H_2_O_2_ to reach the bottom of the hydrogels (Fig. 4a, vial B), making the whole hydrogel lighting up. The slight degradation of the hydrogels was observed to produce some hydrogels fragments, which may speed up the diffusion and mixing process. Alternatively, if the hydrogels were fully mixed with H_2_O_2_ solution at first, CL emission appeared immediately in the entire bulk solution (Fig. 4a, vial A). The diffusion coefficient of H_2_O_2_ in the CS hydrogels was determined, as shown in Supplementary Fig. 12, Table 2 and Note 3. The results demonstrated that the diffusion coefficient of H_2_O_2_ in the CS hydrogels was more than one order of magnitude lower than that in a buffer solution, supporting the slow diffusion of H_2_O_2_ in hydrogels. The superior CL properties of the as-prepared hydrogels are derived from the synergistic effect of ABEI, Co^2+^ and porous network structure of ABEI/Co^2+^/CS hydrogels. It is suggested that Co^2+^ located at the skeleton of CS hydrogels is active site of the CL reaction, which is surrounded by ABEI molecules in pores of the hydrogels. When H_2_O_2_ slowly diffuses to the active site, Co^2+^ as a catalyst would react with H_2_O_2_ to produce a highly reactive hydroxyl radical OH^•^, followed by the reaction with ABEI anion and HO_2_^–^ to facilitate the formation of ABEI radicals and O_2_^•–^. Finally, ABEI radicals react with O_2_^•–^ to generate strong CL emission^15^. Because of the slow diffusion rate of H_2_O_2_ in hydrogels with high viscosity and micro/nano-sized pores, the CL reaction is a slow-diffusion-controlled process and could proceed for several days. Co^2+^ exhibited the strongest catalytic effect for the CL system, which may be due to that Co^2+^ complex demonstrated the strongest decomposition ability of hydrogen peroxide among transition metal ions^23, 24^ and was the best catalyst for luminol and its analogue CL reactions^19^. It was reported that Co^2+^ in the solutions showed very low catalytic activity for the decomposition of H_2_O_2_. Complexation and heterogenization of Co^2+^ enhance the catalytic activity of Co^2+^ on the decomposition of H_2_O_2_^25^. It was also reported that the attachment of catalyst metal complex to a rigid polymer resulted in an increase in the catalytic activity and the stability of catalyst^26, 27^. This is because active site on the polymer was isolated and inactive reactions of catalyst metal ions in the homogeneous phase were prevented^26, 27^. Thus, in this case, Co^2+^ coordinated by hydroxyl and amine groups at the skeleton of CS hydrogels exhibited unique heterogeneous catalytic activity on the CL reaction, leading to intensive emission. The light emission could last for more than 150 h, implying that catalyst Co^2+^ could maintain catalytic activity for a long time. The excellent stability of catalyst Co^2+^ in the hydrogels may be due to the stabilization effect of polymer CS. Accordingly, high efficiency and excellent stability of catalyst Co^2+^ and the slow diffusion rate of H_2_O_2_ in hydrogels with high viscosity and micro/nano-sized pores resulted in the intensive and long-lasting CL emission. It is well known that firefly BL can produce intensive and long-lasting emission. Thus, the intensive and long-lasting CL emission from the hydrogels mimics firefly BL in terms of intensity and duration time. Since Co^2+^ was capable of maintaining catalytic activity for a long time and demonstrated high catalytic efficiency due to the hydrogels, the catalytic characteristic of Co^2+^ in our system are similar to those of enzyme associated with firefly BL^17^.

In conclusion, we have demonstrated firefly-mimicking intensive and long-lasting CL ABEI/Co^2+^/CS hydrogels. The light emission could be observed even by naked eyes in a dark room and lasted for over 150 h when the hydrogels reacted with H_2_O_2_. The intensive and long-lasting CL emission was attributed to the synergistic effect of Co^2+^, ABEI and the porous network structure of the hydrogels through a slow-diffusion-controlled heterogeneous catalytic reaction. Using the low-concentration Co^2+^ as catalyst with high efficiency, CL emission of the as-prepared ABEI/Co^2+^/CS hydrogels mimics firefly BL system in terms of intensity, duration time and catalytic characteristic. Such intensive and long-lasting CL emission with long-lasting mechanism is distinctly different from those of existing enzyme-involved CL and peroxyoxalate ester CL systems. The hydrogels can be used as cold light source in emergency situations, decorative entertainment, and underwater lighting. Compared with the commercial light sources whose emission duration can only reach 10-12 h, our hydrogels achieve a great improvement, which can produce CL emission for over 150 h. Our hydrogels are also environment-friendly and cost-effective. Moreover, the ABEI/Co^2+^/CS hydrogels may find future applications in biosensors, microchips, bioassays and bioimaging, due to the hydrogel’s excellent biocompatibility^28–31^ and intensive and long-lasting CL emission.

## Methods

### Chemicals and materials

A 4.0 mM ABEI stock solution was prepared by dissolving ABEI (TCI, Japan) in 0.1 M NaOH solution. Chitosan (Mv > 1000 kDa, degree of deacytylation > 90%) was obtained from shanghai reagent (Shanghai, China). Working solutions of H_2_O_2_ were prepared fresh daily from 30% (v/v) H_2_O_2_ (Xin Ke Electrochemical Reagent Factory, Bengbu, China). All other reagents were of analytical grade. Ultrapure water was prepared by a Milli-Q system (Millipore, France) and used throughout. All glassware used in the following procedures was cleaned in a bath of freshly prepared HNO_3_-HCl (3:1, v/v), rinsed thoroughly with ultrapure water, and dried prior to use.

### Synthesis of ABEI/Co^2+^/CS hydrogels

Co^2+^/CS hydrogels were synthesized through the freezing-thawing method as previously reported with some modifications^9^. Chitosan powders were dispersed into 15 ml alkaline solution containing LiOH/KOH/urea/H_2_O in a ratio of 4.5: 7: 8: 80.5 by weight. 0.6 ml of CoCl_2_ (1, 5, 10, 20, 30 mM) was added to the above solution with stirring for 5 min, and then were stored under refrigeration until completely frozen. After that, the frozen solid was fully thawed. The Co^2+^/CS hydrogels with 2.5 wt% of chitosan were obtained. Next, 1.5 ml of ABEI (0.04, 0.4, 4 or 40 mM) was added to the Co^2+^/CS hydrogels and stirred for 5 h. Finally, the ABEI/Co^2+^/CS hydrogels were obtained. The as-prepared Co^2+^/CS and ABEI/Co^2+^/CS hydrogels were stored at 4 °C.

### Characterization and property of ABEI/Co^2+^/CS hydrogels

The as-synthesized hydrogels were characterized by SEM, rheological measurements, ICP-AES, inductively coupled plasma mass force microscopy (ICP-MS), UV-visible absorption spectra, microscope imaging, and CL spectra. For the SEM analysis, the Co^2+^/CS hydrogels were diluted 10 times with an alkaline solution containing LiOH/ KOH/urea/H_2_O with a ratio of 4.5: 7: 8: 80.5 by weight. The dilution was refrigerated to form frozen solid, then put into the lyophilizer under a condensing temperature of −50 °C and vacuum degree of 10 Pa. After 48 h, freeze-dried Co^2+^/CS hydrogels were obtained. A thin-layer of freeze-dried Co^2+^/CS hydrogels were deposited onto the conducting film. The morphology of the Co^2+^/CS hydrogels was measured by SEM. SEM images were obtained by a JEOL JEM-6700F microscope (Japan). The porosity of the hydrogels was calculated according to the equation: porosity = [(skeletal density-bulk density)/ skeletal density] × 100%^32^. Specifically, the ABEI/Co^2+^/CS hydrogels were put in a bottle. After freeze-drying process as that in the treatment of the hydrogels for SEM, the weight of freeze-dried ABEI/Co^2+^/CS hydrogels was 0.4122 g, and the volume was 2 cm^3^. Thus, the density of freeze-dried hydrogels (bulk density) was calculated to be 0.2061 g ml^−1^. 1 g of chitosan powders was added to 5 ml of ethanol in the graduated cylinder to obtain a solution with total volume of 5.7 ml (chitosan was not dissoluble in ethanol). Thus, the density of chitosan (skeletal density) was calculated to be 1.4286 g cm^−3^. Porosity could be computed according to above equation. Rheological measurements were conducted on a TA AR-G2 rheometer using a coneplate of 40 mm diameter with a cone angle of 1%. ICP-AES was obtained on an OPTIMA 7000DV atomic emission spectrometer. ICP-MS was measured on PlasmaQuad 3 Thermo VG (UK). UV-visible absorption spectra were obtained using UV-visible spectrophotometer (Agilent 8453, USA). CL spectra were measured on a model F-7000 spectrofluorophotometer when the Xe lamp was turned off (Hitachi, Japan). Fluorescence imaging was obtained on fluorescence microscope (Olympus DP72). CL gel imaging micrograph was performed on ChemiDoc XRS System (BIO-RAD, America). Digital photos were obtained on D7200 Nikon, and every picture was taken by using a delay of 30 s. The static injection CL detection was conducted on a BPCL Luminescence Analyzer (Beijing, China) with a fixed PMT voltage of -550 V. For a typical CL measurement, 100 μl of ABEI/Co^2+^/CS hydrogels was added to a cylindrical cell, then 100 μl of 0.1 M H_2_O_2_ in B-R buffer (pH = 10.88) was injected into the cell to initiate the CL reaction. The CL intensity during the reaction along with time was recorded by the luminescence analyzer.

### Data availability

The authors declare that all the data are available within the article file and its Supplementary Information or from the corresponding author upon reasonable request.

## Electronic supplementary material

Supplementary Information
