# Supplementary material for: Firefly-mimicking intensive and long-lasting chemiluminescence hydrogels
Source: Nat Commun. 2017 Oct 17;8:1003. doi: 10.1038/s41467-017-01101-6 (PMC5645356; doi:10.1038/s41467-017-01101-6)
Supplement: Supplementary file 1 — Supplementary Information [file 41467_2017_1101_MOESM1_ESM.pdf]

Supplementary Information

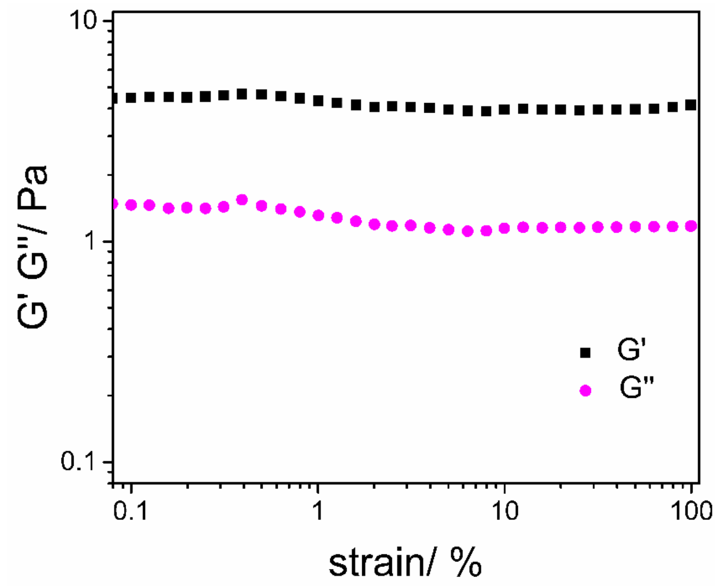

Supplementary Figure 1 | Rheological Property. Dynamic strain of storage modulus (G') and loss modulus (G") of ABEI/Co^{2+}/CS hydrogels at frequency of 1 Hz at 20 °C.

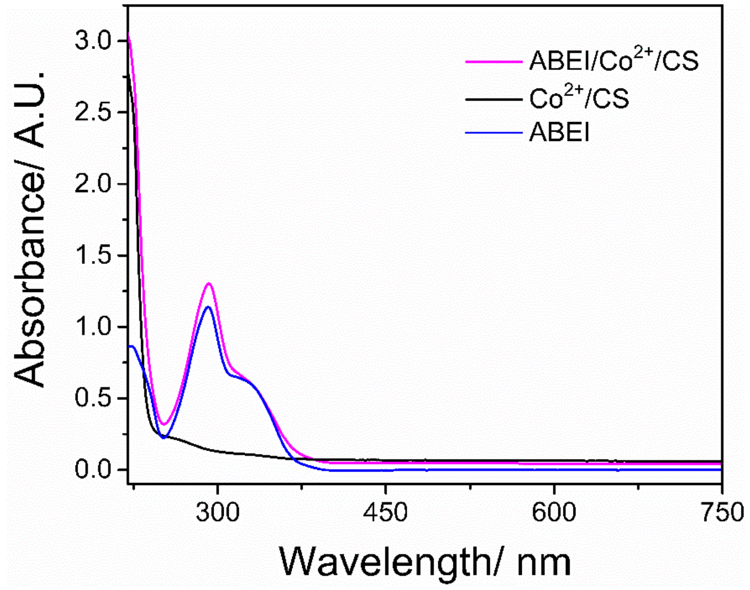

Supplementary Figure 2 | UV-visible absorption spectra of ABEI, Co^{2+}/CS hydrogels and ABEI/Co^{2+}/CS hydrogels.

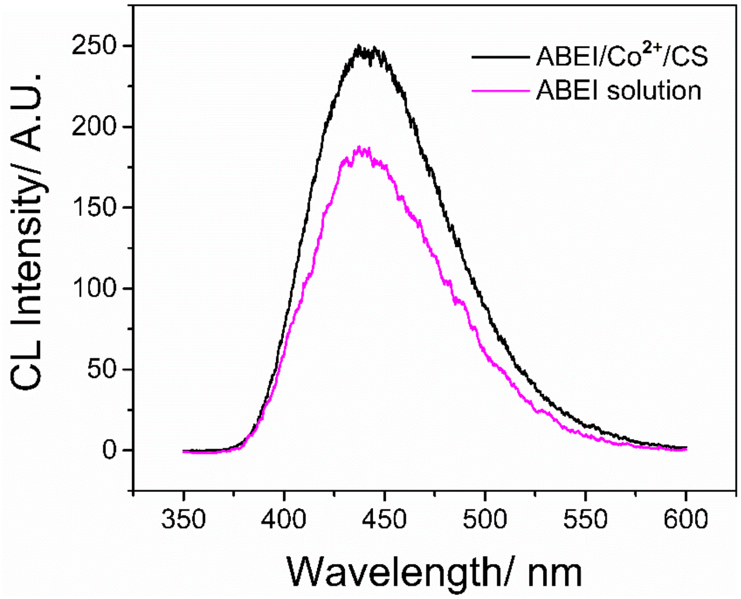

Supplementary Figure 3 | CL spectra of ABEI/Co^{2+}/CS hydrogels and ABEI with H_2O_2.

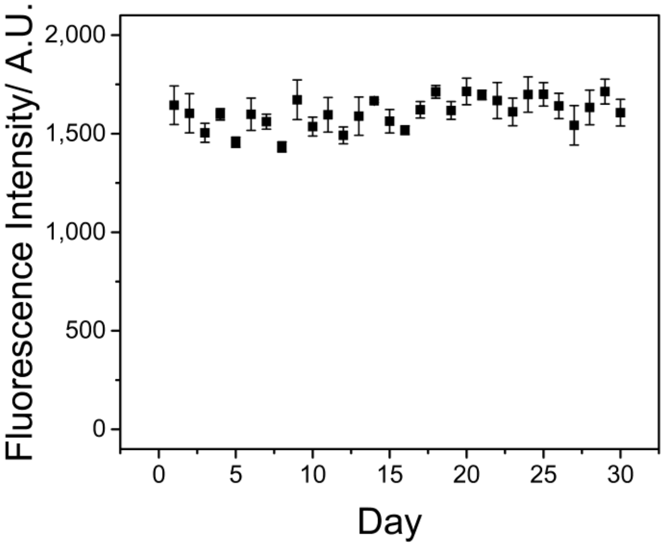

Supplementary Figure 4 | Fluorescence intensity of ABEI/Co^{2+}/CS hydrogels as a function of time over 30 days (n=3, mean ± s.d.). Excitation wavelength: 305 nm, Emission wavelength: 445 nm.

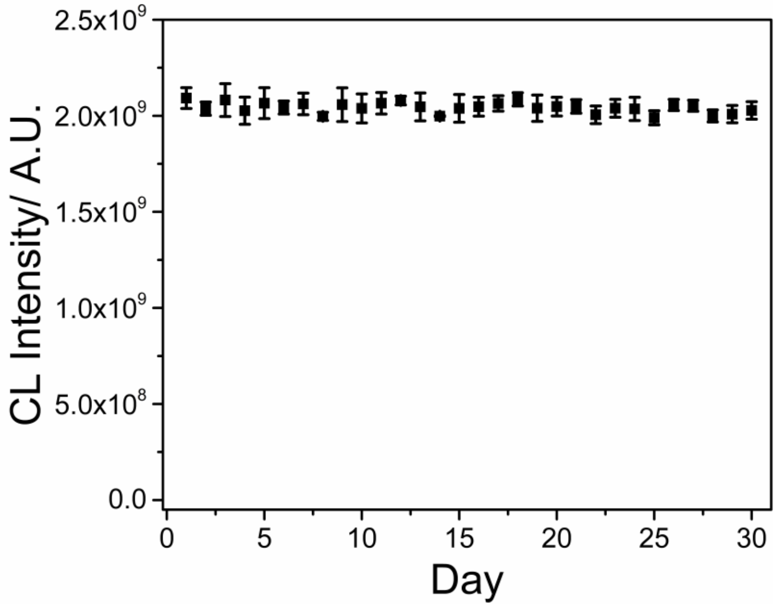

Supplementary Figure 5 | Integrated CL intensity of ABEI/Co^{2+}/CS hydrogels with H_2O_2 in 1 hour over 30 days (n=3, mean ± s.d.).

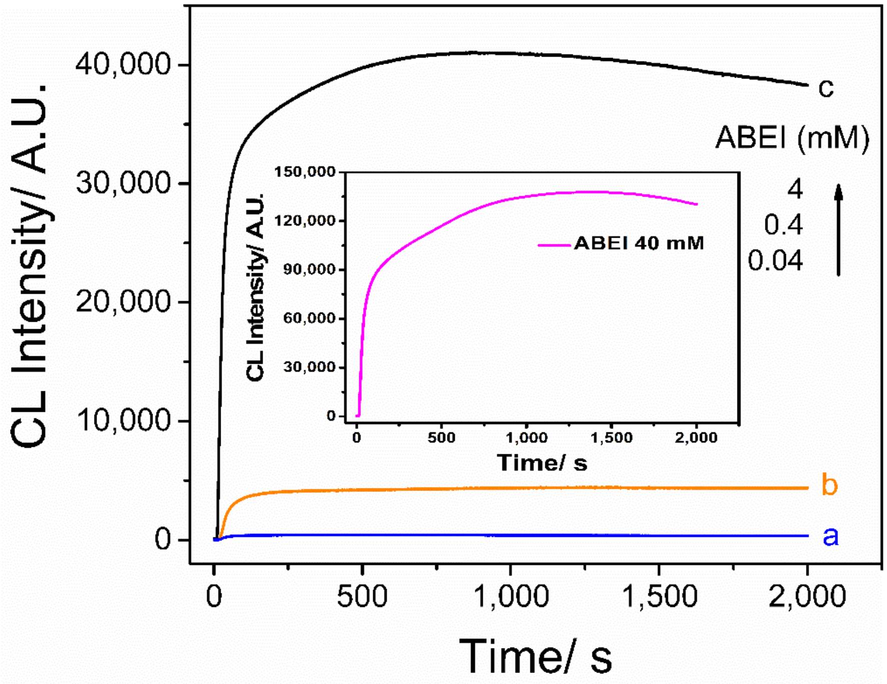

Supplementary Figure 6 | Effect of ABEI concentration on CL emission. CL kinetic curves a-c for reaction of ABEI/Co^{2+}/CS hydrogels at different concentrations of ABEI from 0.04 to 4 mM with H_2O_2. Inset: CL kinetic curve for reaction of ABEI/Co^{2+}/CS hydrogels at 40 mM ABEI with H_2O_2 using a fixed PMT voltage of -500 V (the voltage was decreased due to that the CL intensity would exceed the measuring scale under -550 V). For ABEI/Co^{2+}/CS hydrogels, 1.5 mL ABEI with different concentrations, 10 mM 0.6 mL Co^{2+}, 15 mL CS dispersed in alkaline solution. Reaction condition: 100 μL 0.1 M H_2O_2, 100 μL hydrogels, -550 V PMT.

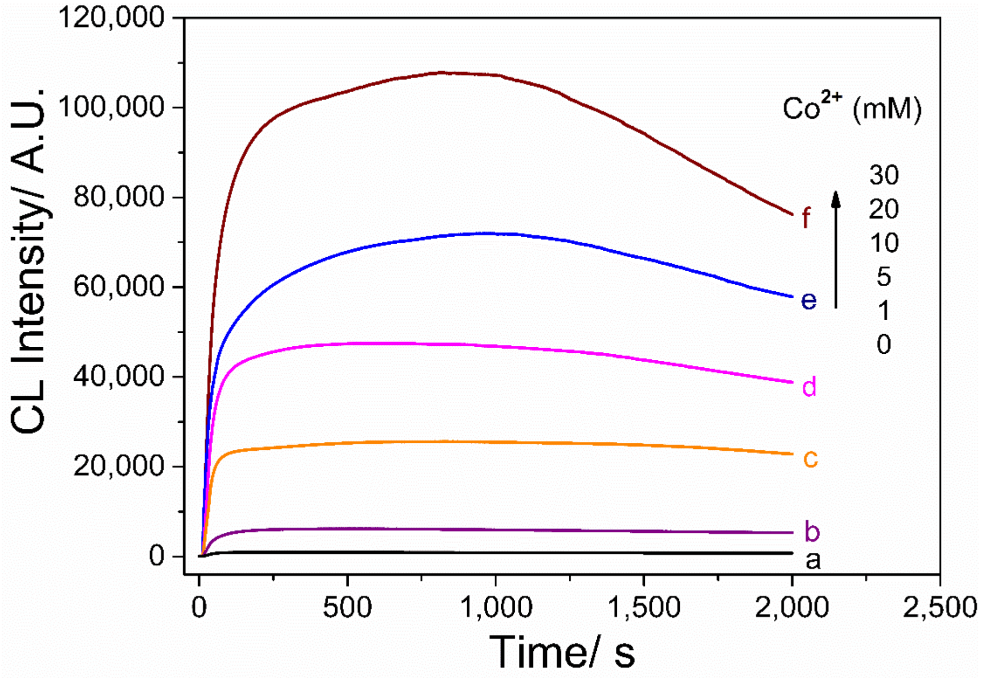

Supplementary Figure 7 | Effect of Co^{2+} concentration on CL emission. CL kinetic curves a-f for reaction of ABEI/Co^{2+}/CS hydrogels at different concentrations of Co^{2+} from 0 to 30 mM with H_2O_2. For ABEI/Co^{2+}/CS hydrogels, 4 mM 1.5 mL ABEI, 0.6 mL Co^{2+} with different concentrations, 15 mL CS dispersed in alkaline solution. Reaction condition: 100 μL 0.1 M H_2O_2, 100 μL hydrogels, -550 V PMT.

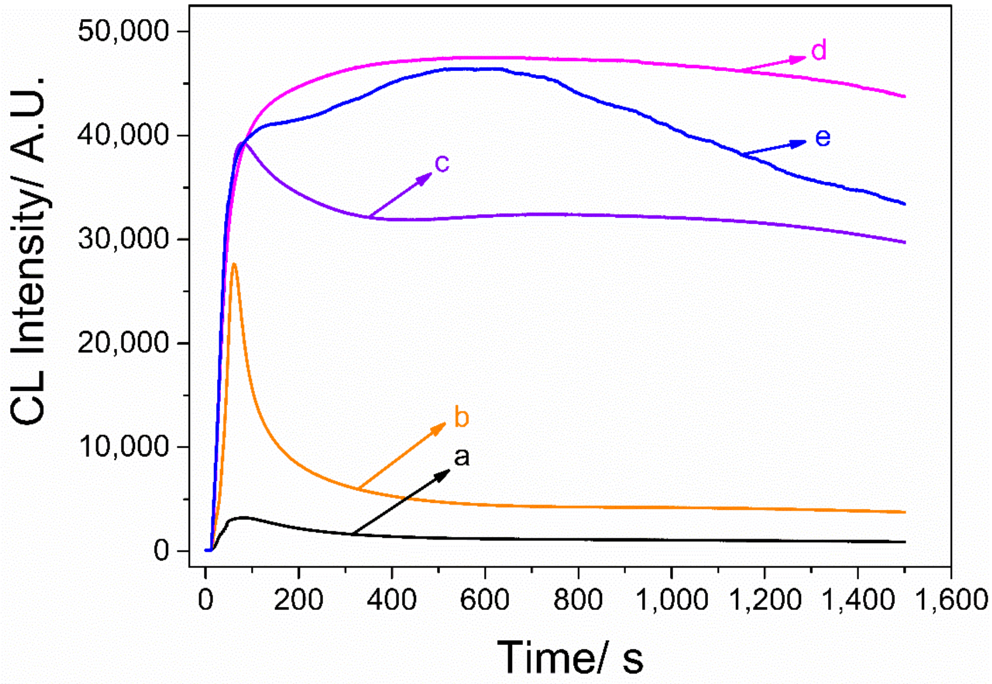

Supplementary Figure 8 | Optimization of H_2O_2 concentration. CL kinetic curves a-e for reaction of ABEI/Co^{2+}/CS hydrogels with different concentrations of H_2O_2 from 0.1-1000 mM. For ABEI/Co^{2+}/CS hydrogels, 4 mM 1.5 mL ABEI, 10 mM 0.6 mL Co^{2+}, 15 mL CS dispersed in alkaline solution. Reaction condition: 100 μL 0.1 M H_2O_2, 100 μL hydrogels, -550 V PMT.

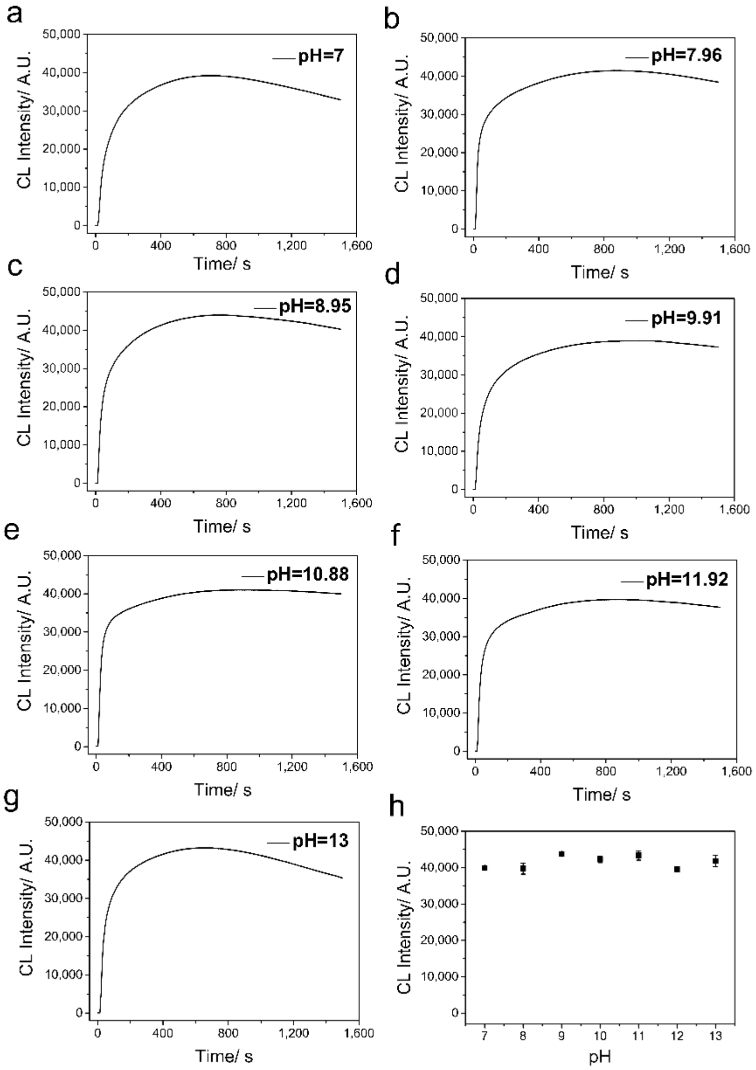

Supplementary Figure 9 | Optimization of pH of H_2O_2. (**a-g**) CL kinetic curves for reaction of ABEI/Co^{2+}/CS hydrogels with H_2O_2 under different pH, wherein H_2O_2 are in B-R buffer (pH=7-11.92) or NaOH solution (pH=13), respectively. (**h**) A comparison of maximal CL intensity of ABEI/Co^{2+}/CS hydrogels reacted with H_2O_2 under different pH as mentioned in a-g. For ABEI/Co^{2+}/CS hydrogels, 4 mM 1.5 mL ABEI, 10 mM 0.6 mL Co^{2+}, 15 mL CS dispersed in alkaline solution. Reaction condition: 100 μL 0.1 M H_2O_2, 100 μL hydrogels, -550 V PMT.

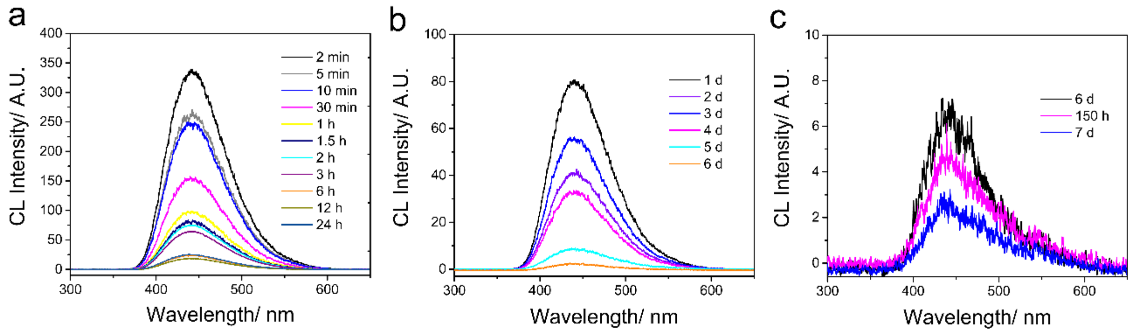

Supplementary Figure 10 | CL spectra of ABEI/Co^{2+}/CS hydrogels at different time. PMT: (**a**) -600 V, (**b**) -700 V, (**c**) -800 V. The CL intensity decreased with time. In order to measure the weak CL intensity, the voltage of PMT must be increased.

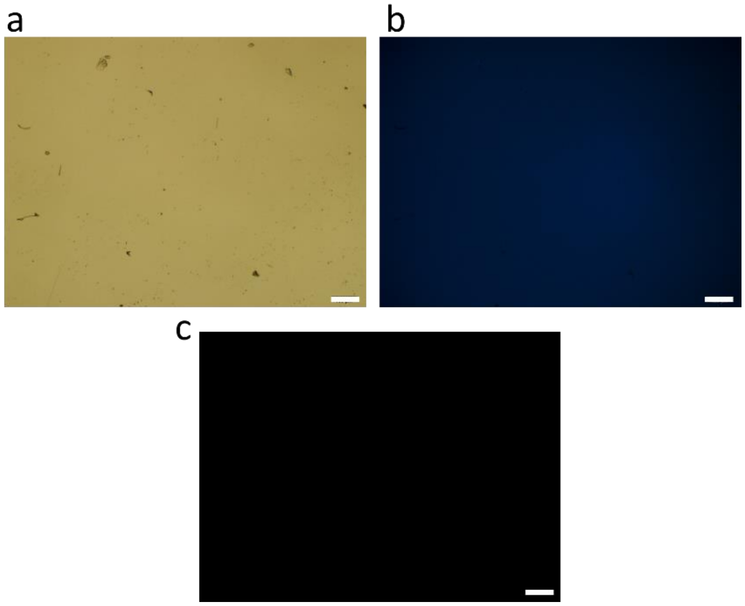

Supplementary Figure 11 | Fluorescence imaging. (**a**) Microimaging photo of ABEI/Co^{2+}/CS hydrogels. (**b**) Fluorescence image (DAPI channel) of ABEI/Co^{2+}/CS hydrogels. (**c**) Blank. The scale bar is 100 µm in all images.

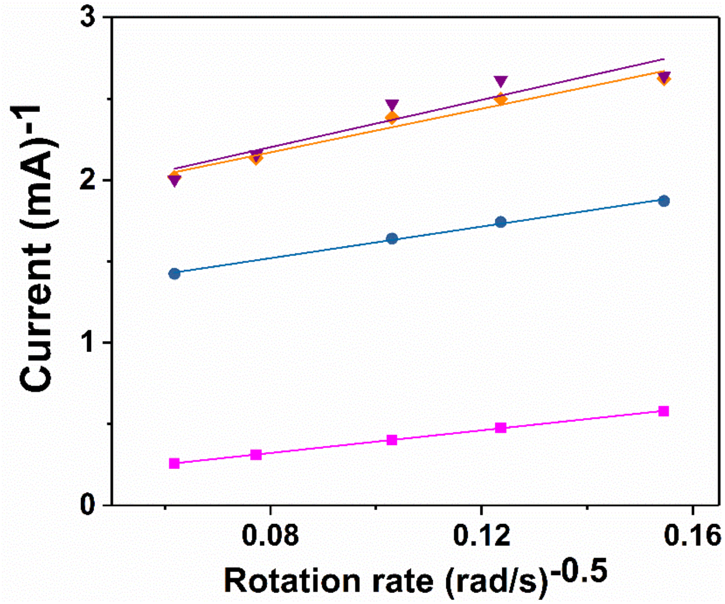

Supplementary Figure 12 | Rotating disc electrode data with a bare Pt electrode (magenta line), CS hydrogel-Pt electrode (blue line), Co^{2+}/CS hydrogel-Pt electrode (orange line) and ABEI/Co^{2+}/CS hydrogel-Pt electrode (purple line) for H_2O_2.[1]

Supplementary Table 1 | Determination of Co^{2+} and ABEI concentration in hydrogel, supernatant and skeleton.

Substance	Hydrogels (mM)	Supernatant (mM)	Skeleton (mM)	Percentage in the aqueous phase	Percentage immobilized at the skeleton
Co^{2+}	$3.51×10^{-2}$	$6.41×10^{-5}$	$3.50×10^{-2}$	0.18%	99.82%
ABEI	3.51	3.14	0.37	89.48%	10.52%

Supplementary Table 2 | Diffusion coefficients in buffer solutions and effective diffusion coefficients in hydrogels for H_2O_2.

T (°C)	BR buffer in this experiment	PBS buffer[1] in the literature	CS hydrogel in this experiment	PVA[a] hydrogel[1] in the literature
	D_{dl}^{exp} (10^{-9} m^2 s^{-1})	D_{dl}^{ref} (10^{-9} m^2 s^{-1})	D_{eff}^{exp} (10^{-9} m^2 s^{-1})	D_{eff}^{ref} (10^{-9} m^2 s^{-1})
25	1.35	1.43	0.113	0.31

[a] Poly(vinyl alcohol)

Supplementary Note 1:

The integrated CL intensity of ABEI/Co^{2+}/CS hydrogels with H_2O_2 as a function of time.

The photo files (.NEF) were converted into images formation (.tif) with 8-bit grayscale LUT. The integrated CL intensity at each photo was obtained by using FIJI software.

Supplementary Note 2:

Determination of Co^{2+} concentration in supernatant

The ABEI/Co^{2+}/CS hydrogels were centrifuged to obtain the supernatant. The concentration of Co^{2+} in the supernatant measured by ICP-MS elemental analysis was 6.41×10^{-5} mM.

Determination of ABEI concentration in supernatant

The ABEI/Co^{2+}/CS hydrogels were centrifuged to obtain the supernatant. The ABEI concentration in the supernatant was determined by the absorbance at 291 nm using spectrophotometry. The ABEI concentration in the supernatant was calculated to be 3.14 mM.

Supplementary Note 3:

Measurement of diffusion coefficients of H_2O_2 in the hydrogels

According to the previous report[1], electrochemical method was conducted to measure the diffusion coefficients of H_2O_2 in the hydrogels. A rotating disc electrode (RDE) covered with hydrogel layer was used to determine the effective diffusion coefficients (D_{eff}) of H_2O_2 in a hydrogel. According to the theory proposed by Tacken and coworkers, the limiting current (I_{limit}) depends on the permeabilities in hydrogel (P_{hl}) and in solution (P_{dl}).

$$\frac{1}{I_{\mathrm{limit}}} = \frac{1}{nFA_{\mathrm{e}}C_{\mathrm{b}}P_{\mathrm{hl}}} + \frac{1}{nFA_{\mathrm{e}}C_{\mathrm{b}}P_{\mathrm{dl}}} \qquad (1)^{1}$$

where n is the number of electrons involved in the electrode reaction, F is the faraday (C), A_{e}, the electrode area (m^2), C_{b} is the bulk concentration (mol m^{-3}).

P_{hl} and P_{dl} are defined by

$$P_{\mathrm{dl}} = {}^{D_{\mathrm{dl}}}\!/_{d_{\mathrm{dl}}} \qquad (2)^{1}$$

and

$$P_{\mathrm{hl}} = {D_{\mathrm{eff}}}/{d_{\mathrm{hl}}} \qquad (3)^{1}$$

where D_{dl} is the diffusion coefficient in solution (m^2 s^{-1}), d_{dl} is the Nernst diffusion layer thickness (m) and d_{hl} is the hydrogel layer thickness (m).

From the theory of mass transfer to an RDE, it is known that

$$d_{\mathrm{dl}} = 1.61\left({D_{\mathrm{dl}}}/{\upsilon_{\mathrm{dl}}}\right)^{1/3}\left({\upsilon_{\mathrm{dl}}}/{\omega}\right)^{1/2} \qquad (4)^{1}$$

where ω is the angular rotation rate (rad s^{-1}) and υ_{dl} is the kinematic viscosity (m^2 s^{-1}). Accordingly, there is linear relationship between the reverse of I_{limit} and the reverse of the square root of $\omega^{-1/2}$.

$$\frac{1}{I_{\mathrm{limit}}} = \frac{1}{nFA_{\mathrm{e}}C_{\mathrm{b}}}\cdot\frac{d_{\mathrm{hl}}}{D_{\mathrm{eff}}} + \frac{1.61\upsilon_{\mathrm{dl}}^{1/6}}{nFA_{\mathrm{e}}C_{\mathrm{b}}}\cdot\frac{1}{D_{\mathrm{dl}}^{2/3}}\cdot\frac{1}{\omega^{1/2}} \qquad (5)$$

Thus, a linear plot can be obtained by plotting the reverse of I_{limit} with the reverse of the square root of $\omega^{-1/2}$. The slope and the intercept of the linear plot give information about D_{dl} in the solution and D_{eff} in the hydrogel layer, respectively.

All electrochemical experiments were conducted on a RDE (Pine Instrument) equipped with a CHI 760E electrochemical workstation (Chenhua, China). A conventional three-electrode assembly was used throughout, consisting of a polished platinum disc (Pt) electrode as the working electrode (A_{e}=5$\times$10^{-9} m^2), Ag/AgCl reference electrode and platinum wire counter electrode. Supporting electrolyte was 0.2 M BR buffer solution (pH 10.88). For the measurement of D_{dl} of H_2O_2 in the buffer solution, Pt electrode was directly used. For the measurement of D_{eff} of H_2O_2 in the hydrogel, 5 µL hydrogel was coated onto the Pt electrode and dried for 1 hour at 60°C. The thicknesses of a swollen gel layer (after

contact with an aqueous solution) on electrode was estimated by dropping 5 μL hydrogel on glass plates with the same area as the Pt electrode (A_e=5×10^{-9} m^2) and the thicknesses of the swollen gel layer was measured with profilometer.

For electrochemical measurements, 10 mol m^{-3} H_2O_2 in 0.2 M BR buffer solution was saturated with argon before voltammograms were scanned from +300 to -800 mV *vs*. Ag/AgCl. The rotation speed for both the Pt electrode and hydrogel-Pt electrodes experiments varied between 1 and 7 s^{-1}.

As shown Supplementary Fig. 12, plots of I_{limit} versus $\omega^{-1/2}$ were linear for measurements with both Pt electrode and hydrogel-Pt electrodes. Table 2 shows the D_{dl} of H_2O_2 in the buffer solution and the D_{eff} of H_2O_2 in the hydrogel layer for various hydrogels (CS hydrogels, Co^{2+}/CS hydrogels and ABEI/Co^{2+}/CS hydrogels).

Supplementary Reference

1. van Stroe-Blezen, S. A. M., Everaerts, F. M., Janssen, L. J. J. & Tacken, R. A. Diffusion coefficients of oxygen, hydrogen peroxide and glucose in a hydrogel. *Anal. Chim. Acta,* **273,** 553-560 (1993).
